# Leptin, Acting at Central Level, Increases FGF21 Expression in White Adipose Tissue via PPARβ/δ

**DOI:** 10.3390/ijms22094624

**Published:** 2021-04-28

**Authors:** Lorena Mazuecos, Cristina Pintado, Blanca Rubio, Eduardo Guisantes-Batán, Antonio Andrés, Nilda Gallardo

**Affiliations:** 1 Regional Center for Biomedical Research (CRIB), University of Castilla-La Mancha, 13071 Ciudad Real, Spain; Lorena.Mazuecos@uclm.es (L.M.); Cristina.Pintado@uclm.es (C.P.); Blanca.Rubio@uclm.es (B.R.); 2 Biochemistry Section, Faculty of Science and Chemical Technologies, University of Castilla-La Mancha, Avda Camilo José Cela 10, 13071 Ciudad Real, Spain; 3 Biochemistry Section, Faculty of Environmental Sciences and Biochemistry, University of Castilla-La Mancha, Avda. Carlos III s/n, 45071 Toledo, Spain; 4 Regional Institute for Applied Scientific Research, University of Castilla-La Mancha, 13071 Ciudad Real, Spain; Eduardo.Guisantes@uclm.es

**Keywords:** epidydimal adipose tissue, FGF21, leptin, PPARβ/δ

## Abstract

The altered function of adipose tissue can result in obesity, insulin resistance, and its metabolic complications. Leptin, acting on the central nervous system, modifies the composition and function of adipose tissue. To date, the molecular changes that occur in epididymal white adipose tissue (eWAT) during chronic leptin treatment are not fully understood. Herein we aimed to address whether PPARβ/δ could mediate the metabolic actions induced by leptin in eWAT. To this end, male 3-month-old Wistar rats, infused intracerebroventricularly (icv) with leptin (0.2 μg/day) for 7 days, were daily co-treated intraperitoneally (ip) without or with the specific PPARβ/δ receptor antagonist GSK0660 (1 mg/kg/day). In parallel, we also administered GSK0660 to control rats fed *ad libitum* without leptin infusion. Leptin, acting at central level, prevented the starvation-induced increase in circulating levels of FGF21, while induced markedly the endogenous expression of FGF21 and browning markers of eWAT. Interestingly, GSK0660 abolished the anorectic effects induced by icv leptin leading to increased visceral fat mass and reduced browning capacity. In addition, the pharmacological inhibition of PPARβ/δ alters the immunomodulatory actions of central leptin on eWAT. In summary, our results demonstrate that PPARβ/δ is involved in the up-regulation of FGF21 expression induced by leptin in visceral adipose tissue.

## 1. Introduction

Leptin is mainly synthesized and secreted by white adipose tissue in proportion to fat content, acting as an energy store signal. Once in circulation, it reaches hypothalamic centers and controls food intake, energy homeostasis, body fat, and immune response by altering the secretion of hypothalamic neuropeptides [1,2,3,4]. In addition, leptin effectively regulates glucose and lipid homeostasis, metabolic effects that occur regardless of their ability to reduce body weight and food intake [5]. Acting through the sympathetic nervous system (SNS) and the neuroendocrine hypothalamic–pituitary–thyroid (HPT) axis, leptin increases lipolysis and promotes adaptive thermogenesis in white adipose tissue, a fact that leads to an increase in energy expenditure and decrease fat stores [6,7,8]. In fact, chronic leptin treatment corrects hyperphagia, impaired lipid metabolism, and thermogenesis in *ob/ob* mice [9].

Moreover, the long-term thermogenic and lipolytic effects of leptin during chronic treatment is achieved through hypothalamic regulation of sympathetic innervation of both brown and white adipose tissue [10]. Specifically, disruption of leptin signalling in the entire arcuate (ARC) leads to a loss of sympathetic innervation of both types of adipose tissues in normal adult mice [10].

In addition, in white adipose tissue, depending on the activation of POMC-positive hypothalamic neurons and the SNS, it has been widely reported that leptin (combined with insulin action) has the ability to promote the browning process as part of a metabolic reprogramming and nutritional adaptation of adipocytes, increasing Ucp-1 expression and other browning markers as Pgc-1α, Prdm16, or β3-adrenergic receptor included [11,12]. However, whether chronic leptin treatment through the CNS regulates the conversion of white to beige fat is only partly understood.

Mountain evidence suggested that leptin deficiency disturb lipid oxidation but its influence on electron transport system appear to depend on the metabolic feature of tissues. In this regard, it has been described that subcutaneous injection of leptin in ob/ob mice decreased protein levels of certain components of OXPHOS complexes in liver and heart, following by a reduction in basal metabolic rate and mitochondrial volume density [13].

Recently it has been known that both pharmacological and genetic inhibition of OXPHOS in adipocytes significantly improved energy homeostasis and insulin sensitivity in mice, accompanied by a markedly increase in adipocyte secretion of Fibroblast Growth Factor 21 (FGF21) [14], another peripheral signal that affects thermogenic activity in both BAT and WAT.

Member of the fibroblast growth factor family of proteins, FGF21 is rapidly induced and secreted by liver in fasting states and mediates the mechanism of adaptation to starvation and multiple stressors in both humans and rodents. Interestingly, it has been found that other tissues as adipocytes, hypothalamus, or pancreas also express this factor in lower levels [15,16,17] and it has been recently hypothesized that FGF21 might have a further additional function as an autocrine/paracrine hormone [18,19,20].

As leptin, FGF21 secretion is related to weight loss and is acutely expressed after cold stimulation in white adipocytes, promoting the induction of Ucp-1 expression and the browning process in rodents [21,22]. In spite of having been reported that leptin might be involved in FGF21 responsiveness or the opposite [23,24], there are few data about a potential metabolic relationship between leptin and FGF21 in vivo. Thus, relevance of adipose tissue and leptin signaling on FGF21 cascade need to be further investigated.

The transcription factor PPARβ/δ is part of the metabolic reprogramming leaded to thermogenesis and browning in white adipocytes, which upregulation reduces adiposity, improves inflammation, and promotes energy expenditure, protecting against obesity in mice [25]. In fact, it has been proposed as a therapeutic target for the treatment of metabolic syndrome [26]. Interestingly, studies from our laboratory have recently shown that leptin, acting in the brain, has the ability to regulate heart lipid content and cardiac remodeling by PPARβ/δ activity [27,28], revealing the capacity of leptin to control cell reprogramming through PPARβ/δ. However, whether PPARβ/δ is a mediator of the central induced actions of leptin in the brain–visceral fat crosstalk has not been elucidated.

To date, since no in vivo investigations using GSK0660 have determined the role of PPARβ/δ on leptin signaling machinery and metabolic function in visceral WAT, we assessed in vivo the effect of the pharmacological inhibition of PPARβ/δ on the metabolic and inflammatory status of eWAT in response to brain leptin infusion. To this end, untreated or icv leptin-treated rats were injected ip with GSK0660, a potent and selective antagonist widely used for determining the biological roles of PPARβ/δ [29]. Moreover, we have also studied the role of icv leptin administration on the regulation of the endogenous expression of FGF21 in eWAT. Of particular interest was to establish the role of PPARβ/δ in the browning of eWAT when the stimulus applied to induce browning is the chronic central leptin infusion. Induction of browning and immune response was assessed by measuring gene and protein expression of several known markers of browning and immune inflammatory response, such as Ucp1, Pparɣ, Pgc-1α, Pdk4, Prdm16, Tbx15, P2rx5, FGF21, Arg1, and Ccl5 in WAT. Our data suggest that PPARβ/δ is involved in the anorexigenic and adipostatic effects of leptin acting at the CNS in Wistar rats, by modulating the transcriptional adaptation of eWAT to chronic leptin treatment regulation of innate immunity and browning.

## 2. Results

### 2.1. Whole Body Pharmacological Inhibition of PPARβ/δ Reduced the Effects of Central Leptin in PPARβ/δ Expression in eWAT, and Abolished the Anorexigenic and Adipostatic Actions of Leptin

**Figure 1:**
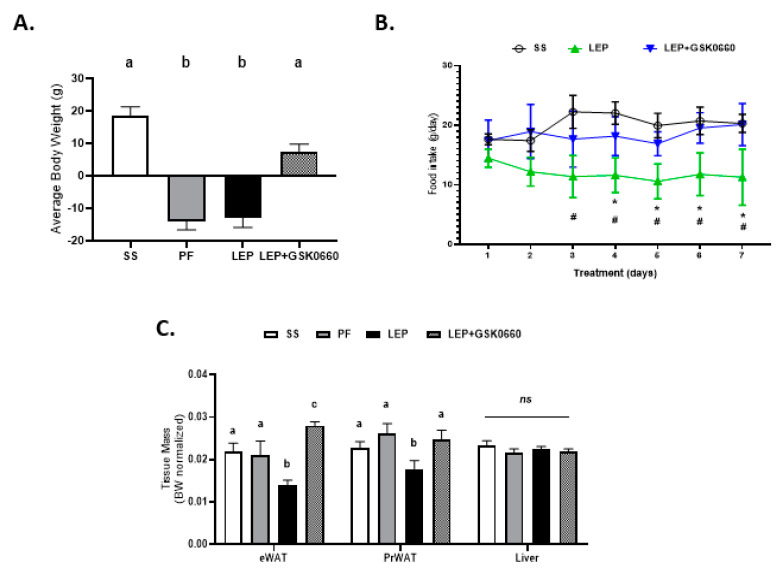

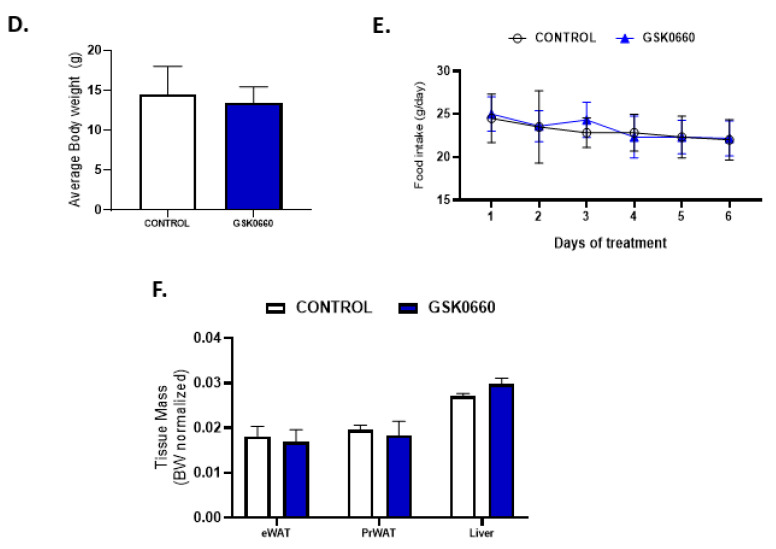
Change in body weight (**A**) and food intake evolution (**B**) of rats after 7 days of chronic central treatment of vehicle (SS and PF), leptin, or leptin and ip GSK0660 co-administration (*n* = 6–8). (**C**) Tissue mass of epididymal white adipose tissue (eWAT), peritoneal white adipose tissue (PrWAT) and liver in rats after 7 days of chronic central treatment of vehicle (SS and PF), leptin, or leptin and ip GSK0660 co-administration (*n* = 6–8) Change in body weight (**D**) and food intake evolution (**E**) of rats after 7 days of vehicle (DMSO) control or antagonist GSK0660 intraperitoneal injection (*n* = 6). (**F**) Tissue mass of epididymal white adipose tissue (eWAT), peritoneal white adipose tissue (PrWAT) and liver in rats after 7 days of vehicle (DMSO) control or antagonist GSK0660 intraperitoneal injection (*n* = 6). Results are the mean ± SEM per group of animals (*n* = 6–8). Differences between CONTROL vs. GSK0660 treatment were analyzed by Student’s *t*-test (* *p* < 0.05; ns: non-significant). Significant differences between ICV-treated rats were analyzed Student’s *t*-test, SS vs. Lep * *p* ≤ 0.05; Lep vs. Lep+GSK0660 # *p* < 0.05). Significant differences between ICV-treated rats were analyzed by One-Way ANOVA followed by Tukey test (differences letters mean significant differences among treatments, *p* ≤ 0.05). ^a^ *p* ≤ 0.05 vs. PF or Lep or Lep+GSK0660; ^b^ *p* ≤ 0.05 vs. SS or PF or Lep+GSK0660; ^c^ *p* ≤ 0.05 vs. SS or PF or Lep. SS: vehicle-infused rats fed ad libitum; PF: vehicle-infused pair-fed rats; Lep: leptin-infused rats; Lep+GSK0660: leptin-infused rats plus PPARβ/δ antagonist GSK0660.

Initially, we wanted to confirm the ability of exogenous central leptin infusion to activate hypothalamic leptin signaling. Thus, we examined the hypothalamic mRNA levels of the long *Ob-Rb* leptin receptor and those of *Pomc, Crh*, and *Trh*, three target genes for the action of leptin, in saline, pair-fed, and leptin treated rats, at the time of sacrifice. As expected, leptin induced hypothalamic *Ob-Rb, Pomc, Crh*, and *Trh* expression compared to the pair-fed rats, consistent with unaltered central leptin sensitivity (Supplemental Table S3). Accordingly, and supporting previous observations [6,30], chronic central leptin infusion notably decreased body weight gain and daily food intake when compared with saline-treated rats (SS) (Figure 1A,B) as well as the epididymal and retroperitoneal adipose tissue mass when compared with pair-fed rats (Figure 1C). Of note, GSK0660 administration in leptin-treated rats caused an opposite effect on body weight, food intake, and adiposity, blunting the anorexigenic and adipostatic effects of central leptin, (Figure 1A–C). Moreover, no changes in liver tissue mass were found (Figure 1C). Therefore, we suggest that the body weight differences found in rats co-treated centrally with leptin and intraperitoneally with GSK0660 are largely due to the differences in visceral fat mass.

**Figure 2:**
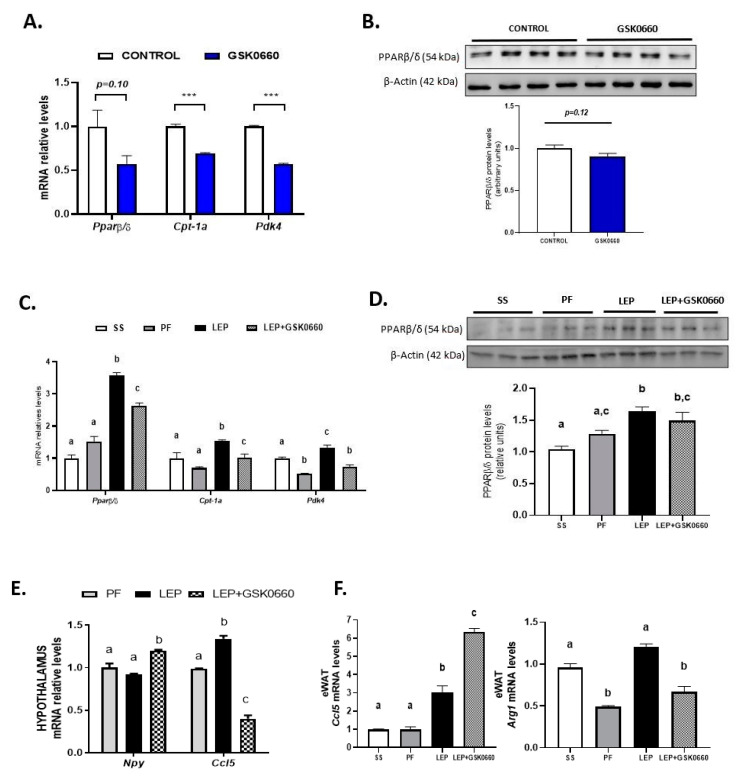
(**A**) Effect of vehicle (DMSO) control or antagonist ip GSK0660 administration on eWAT mRNA levels of *Pparβ/δ*, *Cpt1a,* and *Pdk4* of rats after 7 days of intraperitoneal injection (*n* = 6). (**B**) Representative Western Blot and relative densitometric analysis of PPARβ/δ total protein content in eWAT of rats after 7 days of vehicle (DMSO) control or ip GSK0660 injection (*n* = 6). (**C**) Effect of icv vehicle (SS and PF), leptin, or leptin and ip GSK0060 co-administration on eWAT mRNA levels of *Pparβ/δ*, *Cpt1a* and *Pdk4* in rats after 7 days of chronic treatment (*n* = 4–6). (**D**) Representative Western blot and relative densitometric analysis PPARβ/δ total protein content in eWAT in rats after 7 days of chronic central treatment of vehicle (SS and PF), leptin, or leptin and ip GSK0660 co-administration (*n* = 4–6). (**E**) Hypothalamus mRNA levels of *Npy* and *Ccl5* in rats after 7 days of chronic central treatment of vehicle (SS and PF), leptin, or leptin and ip GSK0660 co-administration. (**F**) eWAT mRNA levels of *Ccl5* and *Arg1* in rats after 7 days of chronic central treatment of vehicle (SS and PF), leptin, or leptin and ip GSK0660 co-administration. Data from real-time RT-PCR are expressed relative to 18S rRNA and the mRNA levels are expressed relative to the control (DMSO) group and vehicle SS group, the level of which was set at 1.0 arbitrarily. In Western blot analysis, densitometric levels of proteins are expressed relative to the control group and SS group, the levels of which were set at 1.0 arbitrarily. Results are the mean ± SEM per group of animals (*n* = 4–6). Differences between CONTROL vs. GSK0660 treatment were analyzed by Student’s *t*-test (*** *p* < 0.001). Significant differences between ICV-treated rats were analyzed by One-Way ANOVA followed by Tukey test (differences letters mean significant differences among treatments, *p* ≤ 0.05). ^a^ *p* ≤ 0.05 vs. Lep or Lep+GSK0660; ^b^ *p* ≤ 0.05 vs. SS or PF or Lep+GSK0660; ^c^ *p* ≤ 0.05 vs. SS or PF or Lep or Lep+GSK0660. SS: vehicle-infused rats fed ad libitum; PF: vehicle-infused pair-fed rats; Lep: leptin-infused rats; Lep+GSK0660: leptin-infused rats plus PPARβ/δ antagonist GSK0660.

In contrast, in non-leptin-treated rats with free access to chow diet, GSK0660 treatment alone had no effect on body weight gain or food intake (Figure 1D,E). In addition, no change was either observed in eWAT, retroperitoneal white adipose tissue (PrWAT), or liver weight upon GSK0660 administration (Figure 1F). Nevertheless, GSK0660 treatment notably increased serum levels of leptin, insulin, and triglyceride in rats, without affecting glucose levels (Table 1), suggesting that GSK0660 induced insulin resistance in 3-month-old Wistar rats. Moreover, whole body inhibition of PPARβ/δ in non-leptin-treated rats caused a significant decrease on the expression of *Cpt-1a* and *Pdk4*, (PPARβ/δ target genes) in eWAT, confirming a partial inhibition of the transcriptional activity of this nuclear receptor (Figure 2A). In fact, *PPARβ/δ* gene expression was also reduced (*p* = 0.1) after GSK0660 injection compared to control group (Figure 2A), although protein levels were not affected (Figure 2B). These data indicate that PPARβ/δ might regulate its own gene expression in eWAT from Wistar rats.

As central leptin increased *PPARβ/δ* expression in heart [27], we next analyzed whether leptin was able to upregulate the expression of *PPARβ/δ* in eWAT. Central chronic infusion of leptin increased *PPARβ/δ* gene expression by 3.5-fold in eWAT compared with pair-fed rats (Figure 2C). Furthermore, the protein levels of this transcription factor were also markedly increased (Figure 2D). As expected, mRNA levels of *Cpt-1a* and *Pdk4* were also increased parallel to PPARβ/δ induction and independently of the anorexigenic effects of leptin, confirming an increase in oxidative capacity of eWAT (Figure 2C). According to Figure 2A, the pharmacological inhibition of PPARβ/δ during leptin treatment reduced *PPARβ/δ*, *Cpt-1a,* and *Pdk4* gene expression (Figure 2C), without affecting PPARβ/δ protein levels (Figure 2D).

In accordance with our previous results [6,30], 7 days chronic central leptin administration at a very low dose decreased serum insulin levels in 3-month-old rats, very likely due to its capacity to suppress insulin secretion and to enhance insulin sensitivity through central leptin-mediated activation of the SNS (Table 1). Interestingly, when leptin and GSK0660 are co-administered, brain leptin maintains its ability to regulate insulin secretion and glucose levels, despite the whole body-inhibition of PPARβ/δ receptor (Table 1).

Total cholesterol was reduced in pair-fed and leptin-treated rats, and according to its lipostatic function, leptin also reduced serum levels of triglycerides (Table 1). No notable changes were observed in leptin-GSK0660 co-treated rats in cholesterol levels when compared with pair-fed or leptin groups (Table 1). Interestingly, PPARβ/δ antagonist administration increased triglyceride serum levels in leptin- and non-leptin-treated rats (Table 1), which could be associated to downregulation of *Cpt1a*, at least, in eWAT (Figure 2A,C). These results confirm that GSK0660 reduced the positive impact of PPARβ/δ on fatty acid oxidation [29].

Leptin infusion tended to decrease serum leptin levels in leptin-treated rats, but this tendency was not statistically significant. Moreover, there was an increase, albeit not statistically significant at *p* ≤ 0.05, in leptin levels after inhibition of PPARβ/δ in leptin-treated rats (*p* = 0.08 Lep+GSK0660 vs. Lep) (Table 1), in parallel with the increase in eWAT (Figure 1C). These data allow us to suggest that leptin sensitivity might be impaired by GSK0660.

### 2.2. Changes in the Immunomodulatory Actions of Leptin in Hypothalamus and eWAT upon PPARβ/δ Inhibition

To identify mechanisms by which GSK0660 interfere in the anorexigenic and adipostatic effects of leptin in rats, we measured in the hypothalamus the expression of *Npy* as well as *Ccl5*, a chemokine implicated in the hypothalamic regulation of food intake and insulin-related energy homeostasis [31]. As seen in Figure 2E, treatment with GSK0660 changes the effects of leptin on Npy and Ccl5 (RANTES) expression, which is indicative that the actions of leptin at the hypothalamic level go through the activation of PPARβ/δ, which could result in the control of energy intake and expenditure confirming a previous role assigned to neuronal PPARβ/δ reported in neuron-specific PPARβ/δ deficient mice [32].

We further analyzed in eWAT the expression of *Arg1*, a hallmark of alternatively activated macrophages and *Ccl5* given its role in local inflammation of visceral WAT in obesity. Importantly, leptin-GSK0660 co-treatment dramatically reduced *Arg1*, while increased *Ccl5* mRNA levels in this group of rats compared to leptin-treated rats (Figure 2F). Although we do not know what the source of *Ccl5* is, these data might suggest that increased adiposity could be accompanied by immune cells (macrophages and T cells) infiltration, reduction of alternatively activated macrophages and local tissue inflammation in eWAT [33].

### 2.3. Pharmacological Inhibition of PPARβ/δ Affects Leptin Signaling in eWAT

**Figure 3:**
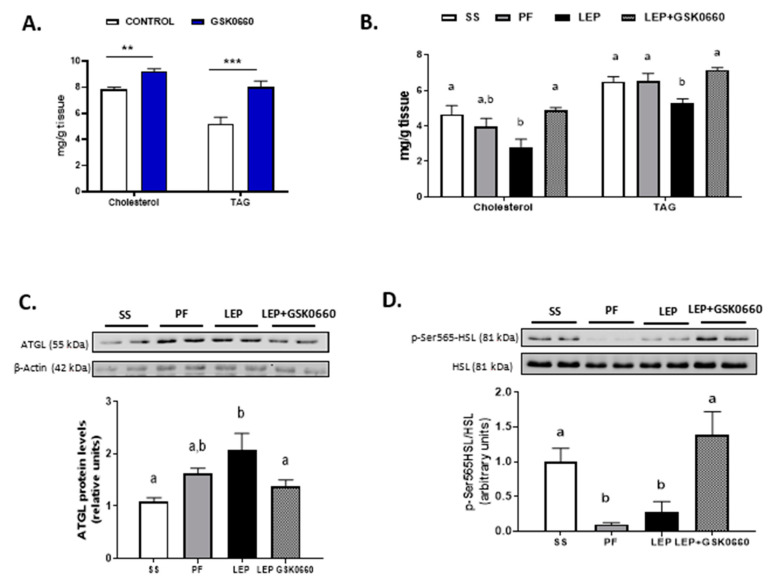
(**A**) Cholesterol and triglyceride (TAG) levels in eWAT in rats after 7 days of vehicle (DMSO) control or antagonist GSK0660 intraperitoneal injection (*n* = 6). (**B**) Cholesterol and triglyceride levels in eWAT in rats after 7 days of chronic central treatment of vehicle (SS and PF), leptin, or leptin and ip GSK0660 co-administration (*n* = 4–6). (**C**) Representative Western Blot and relative densitometric analysis of ATGL total protein content in eWAT after 7 days of chronic central treatment of vehicle (SS and PF), leptin, or leptin and ip GSK0660 co-administration (*n* = 4–6). (**D**) Representative Western blot of p-SER565-HSL and total HSL protein levels and relative densitometric analysis of the ratio p-Ser565HSL/HSL in eWAT of rats after 7 days of chronic central treatment of vehicle, leptin, or leptin and GSK0660 co-administration (*n* = 4–6). Results are the mean ± SEM per group of animals (*n* = 4–6). Densitometric levels of proteins are expressed relative to the SS group the level of which was set at 1.0 arbitrarily. Differences between CONTROL vs. GSK0660 treatment were analyzed by Student’s *t*-test (** *p* < 0.01, *** *p* < 0.0001). Significant differences between ICV-treated rats were analyzed by One-Way ANOVA followed by Tukey test (differences letters mean significant differences among treatments, *p* ≤ 0.05). ^a^ *p* ≤ 0.05 vs. PF or Lep; ^b^ *p* ≤ 0.05 vs. SS or PF or Lep+GSK0660. SS: vehicle-infused rats fed ad libitum, PF: vehicle-infused pair-fed rats; Lep: leptin-infused rats; Lep+GSK0660: leptin-infused rats plus PPARβ/δ antagonist GSK0660.

Because the role of PPARβ/δ in WAT leptin metabolism is unknown, we further investigate the molecular changes in eWAT related to leptin signaling. Consistent with the increased circulating levels of leptin in non-leptin-treated rats (Table 1), we found that GSK0660 administration increased *leptin* and *Ob-Rb* mRNA levels in eWAT (Table 2), indicating a state of energy sufficiency. In fact, GSK0660 treatment remarkably increased triglyceride and cholesterol content in eWAT (Figure 3A), as it has been reported in myotubes, macrophages, fibroblasts, and intestinal cells [34,35,36].

Leptin infusion reduced the expression of *leptin* gene in eWAT independently of its anorectic effects and the metabolic effects of GSK0660 (Table 2). Although not clear, it has been recently suggested that the mRNA levels of *leptin* strongly correlate with fat mass content [37,38]. On the contrary, although leptin infusion did not affect gene expression of *Ob-Rb* in eWAT compared to the pair-fed rats, when leptin and GSK0660 were co-administered, the mRNA levels of *Ob-Rb* were markedly reduced (Table 2), which diminishes the potential of leptin to directly influence the metabolism of adipocytes. This result is in line with previous finding indicating that caloric restriction enhances *Ob-Rb* expression and leptin responsiveness in leptin-resistant DIO rats; thus, levels of *Ob-Rb* depend on food intake [39].

Moreover, and supporting prior data [6], central leptin reduced both the cholesterol and triglyceride content in eWAT (Figure 3B). In contrast, the pharmacological inhibition of PPARβ/δ abolished the effects of central leptin, increasing cholesterol and triglyceride levels in GSK0660-leptin co-treated rats (Figure 3B). Hence, we hypothesized that differences in triglyceride content could be linked to disturbed adipocyte lipolysis in leptin-GSK0660 co-treated rats. Interestingly, we found that GSK0660 could reduce the lipolytic effects induced by central leptin in eWAT by lowering ATGL protein levels (Figure 3C) and increasing HSL-Ser565 phosphorylation levels, which abolishes cyclic AMP-dependent protein kinase (PKA)-induced HSL activation (Figure 3D). All these results suggest an impairing in central leptin signalling transduction and PKA-mediated induction of lipolysis in eWAT upon PPARβ/δ inactivation.

### 2.4. Enhancement of β-Klotho Protein Levels in eWAT by Both icv Leptin, or Leptin and ip GSK0660 co-Treated Rats

Fibroblast Growth Factor 21 (FGF21) has been described as a potent metabolic regulator with anti-diabetic properties, but still a complex hormone with unknown functions in multiple target tissues [19,40]. Hepatic FGF21 is a negative regulator of adipose tissue lipolysis during fasting [41], while FGF21 locally released by 3T3-adipocytes, after β-adrenergic stimulation, induces insulin-independent glucose uptake in these cells through an autocrine mechanism [20].

**Figure 4:**
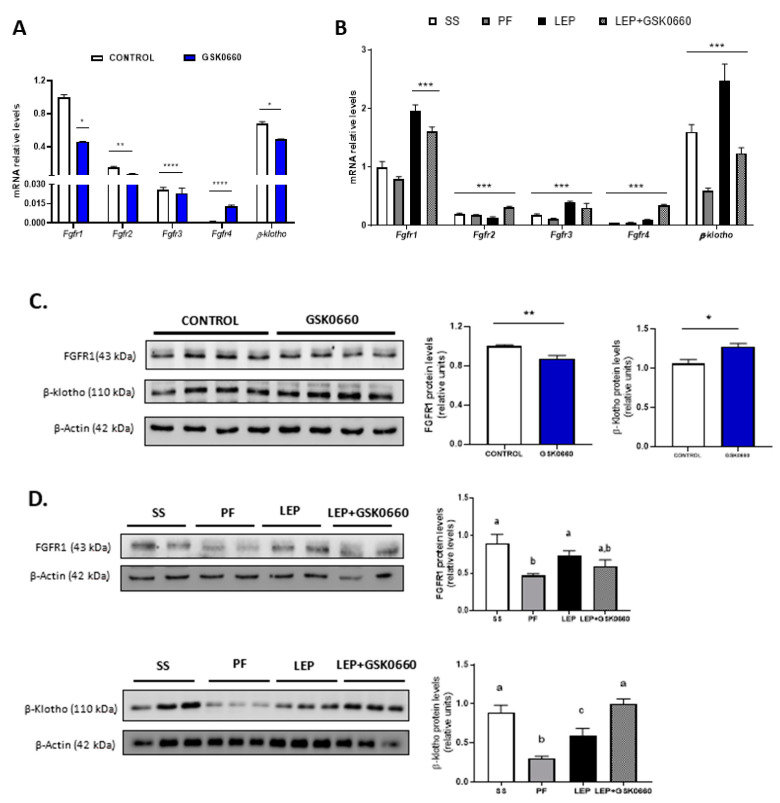
(**A**) Quantification and gene expression comparison between FGFR1-4 and β-klotho co-receptor in eWAT of rats after 7 days of vehicle (DMSO) control or intraperitoneal GSK0660 injection (*n* = 6). (**B**) Gene expression comparison between FGF receptors and β-klotho co-receptor in eWAT of rats after 7 days of chronic central treatment of vehicle (SS and PF), leptin, or leptin and ip GSK0660 co-administration (*n* = 4–6). (**C**) Representative Western blot and relative densitometric analysis of FGFR1 and β-klotho total protein content in eWAT of rats after 7 days of vehicle (DMSO) control or intraperitoneal GSK0660 injection (*n* = 6). (**D**) Representative Western blot and relative densitometric analysis of FGFR1 and β-klotho total protein content in eWAT of rats after 7 days of chronic central treatment of vehicle (SS and PF), leptin, or leptin and ip GSK0660 co-administration (*n* = 4–6). Data from real-time RT-PCR are expressed relative to 18S rRNA and the mRNA levels are expressed relative to the Fgfr1 gene expression of Control group and SS group, the level of which were set at 1.0 arbitrarily. Results are mean ± S.E.M (*n* = 4–6) and were analyzed by Nested one-way ANOVA followed by Dunnett’s test (* *p* < 0.05, ** *p* < 0.01, *** *p* < 0.001, **** *p* < 0.0001 vs. Fgfr1 gene expression). In Western blot analysis, densitometric levels of proteins are expressed relative to the Control group and SS group, the levels of which were set at 1.0 arbitrarily. Results are the mean ± SEM per group of animals (*n* = 4–6). Differences between CONTROL vs. GSK0660 treatment were analyzed by Student’s *t*-test (* *p* < 0.05, ** *p* < 0.001). Significant differences between ICV-treated rats were analyzed by One-Way ANOVA followed by Tukey test (differences letters mean significant differences among treatments, *p* ≤ 0.05). ^a^ *p* ≤ 0.05 vs. PF or Lep; ^b^ *p* ≤ 0.05 vs. SS or Lep or Lep+GSK0660; ^c^ *p* ≤ 0.05 vs. SS or PF or Lep+GSK0660. SS: vehicle-infused rats fed ad libitum, PF: vehicle-infused pair-fed rats; Lep: leptin-infused rats; Lep+GSK0660: leptin-infused rats plus PPARβ/δ antagonist GSK0660.

**Figure 5:**
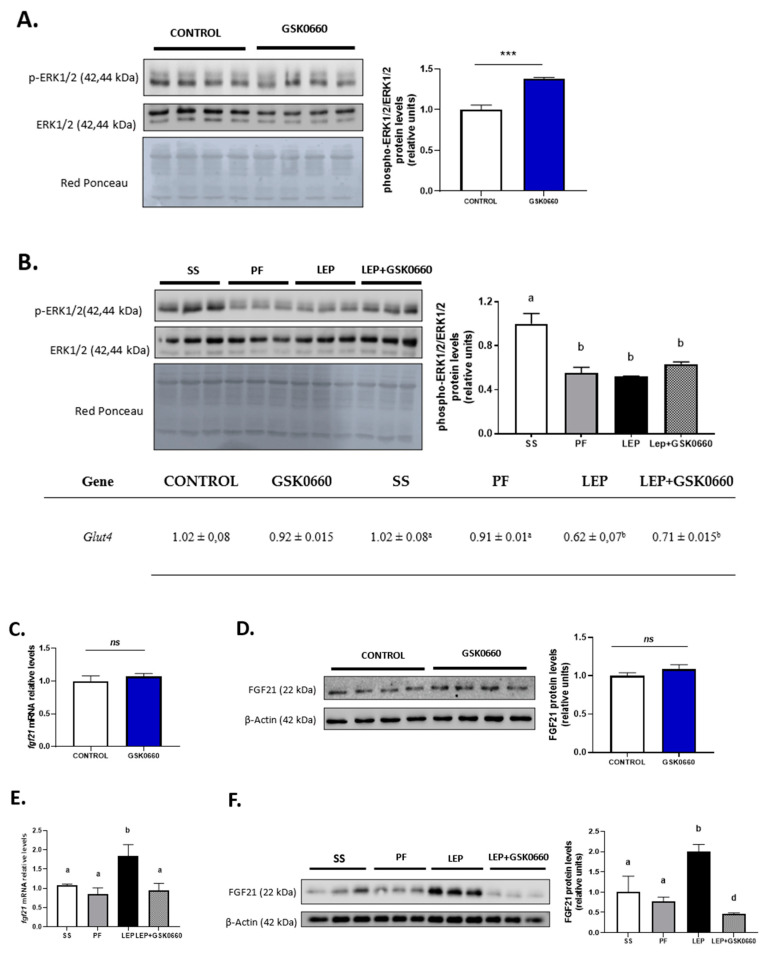
(**A**) Representative Western Blot of p-ERK1/2 and total ERK1/2 protein levels and relative densitometric analysis of the ratio p-ERK1/2/ERK1/2 in eWAT of rats after 7 days of vehicle (DMSO) control or intraperitoneal antagonist GSK0660 injection (*n* = 6). (**B**) mRNA levels of Glut4, representative Western Blot of p-ERK1/2 and total ERK1/2 protein levels and relative densitometric analysis of the ratio p-ERK1/2/ERK1/2 in eWAT of rats after 7 days of chronic central treatment of vehicle (SS and PF), leptin, or leptin and ip GSK0660 co-administration (*n* = 4–6). Effect of vehicle (DMSO) control or ip antagonist GSK0660 administration on eWAT mRNA levels (**C**) and protein levels (**D**) of FGF21 in rats after 7 days injection (*n* = 6). Effect of icv vehicle (SS and PF), leptin, or leptin and ip GSK0060 co-administration on eWAT mRNA levels (**E**) and protein levels (**F**) of FGF21 in rats after 7 days of chronic treatment (*n* = 4–6). Data from real-time RT-PCR are expressed relative to 18S rRNA and the mRNA levels are expressed relative to the Control group and SS group, the level of which was set at 1.0 arbitrarily. In Western blot analysis, densitometric levels of proteins are expressed relative to the Control group and SS group, the levels of which were set at 1.0 arbitrarily. Results are the mean ± SEM per group of animals (*n* = 4–6). Differences between CONTROL vs. GSK0660 treatment were analyzed by Student’s *t*-test (*** *p* < 0.001). Significant differences between ICV-treated rats were analyzed by One-Way ANOVA followed by Tukey test (differences letters mean significant differences among treatments, *p* ≤ 0.05). ^a^ *p* ≤ 0.05 vs. Lep or Lep+GSK0660; ^b^ *p* ≤ 0.05 vs. SS or PF or Lep+GSK0660; ^c^ *p* ≤ 0.05 vs. SS or PF or Lep. SS: vehicle-infused rats fed ad libitum, PF: vehicle-infused pair-fed rats; Lep: leptin-infused rats; Lep+GSK0660: leptin-infused rats plus PPARβ/δ antagonist GSK0660.

As shown in Table 1, in non-leptin-treated rats antagonist GSK0660 administration alone did no change circulating levels of FGF21, while reduced mRNA levels of *β-klotho* and *Fgfr1*, the main protein receptors of this family expressed in eWAT (Figure 4A). In accordance, FGFR1 protein levels were decreased after GSK0660 administration (Figure 4C). Nevertheless, β-klotho protein levels were increased in eWAT after GSK0660 treatment (Figure 4C). As FGF21/FGFR1 cascade phosphorylates the extracellular signal-regulated kinases 1 and 2 (ERK1/2) and regulates *Glut4* expression, we further evaluate the ratio phosphorylated ERK1/2 to total ERK1/2 and the mRNA levels of *Glut4* (also a target of ERK1/2) in eWAT at basal levels. Although the basal levels of both p-ERK and total ERK were decreased after inhibition of PPARβ/δ receptor (* *p* = 0.048, ** *p* = 0.003, respectively, Student’s *t* test) (Figure 5A), the pharmacological inhibition of PPARβ/δ with GSK0660 increased the ratio p-ERK/ERK in eWAT (Figure 5A).

Despite not having changes in liver FGF21 protein levels (Supplemental Figure S1), leptin infusion markedly reduced serum levels of the hormone in both leptin-treated group of rats, comparing with its pair-fed control group (Table 1), in which FGF21 levels were acutely increased very likely due to fasting and caloric-restricted state. Inhibition of PPARβ/δ did not apparently affect FGF21 circulating levels when it was co-administered with leptin (Table 1). Thus, the effects of leptin on circulating FGF21 are not dependent on PPARβ/δ activity. Upon central administration of leptin for 7 days, the mRNA and protein levels of FGFR1 and β-klotho were notably induced in eWAT when compared to its pair-fed treated rats (Figure 4B,D). Moreover, the pharmacological inhibition of PPARβ/δ in leptin-treated rats decreased mRNA levels of both FGFR1 and β-klotho in leptin-treated rats (Figure 4B). Unexpectedly, the inhibition of PPARβ/δ did not reduce FGFR1 and β-klotho protein levels in eWAT (Figure 4D).

As indicated above, GSK0660 administration did not alter serum FGF21 levels in leptin-treated rats. However, FGFR1 protein levels and *Fgfr1* and *β-klotho* expression were downregulated in these rats (Figure 4B,D). As shown in Figure 5B, basal phosphorylated state of ERK1/2 was also reduced compared to saline-infused ad libitum fed rats (SS), suggesting a decrease in eWAT proteins from FGF21 signaling cascade during caloric restriction. Interestingly, when leptin was centrally infused, circulating levels of FGF21 notably decreased in these rats, independently of the anorexigenic effects of leptin, while both FGFR1 and β-klotho gene expression and protein levels were upregulated. Furthermore, the stage of the activated form of ERK1/2 is maintained similar to levels found in pair-fed rats (Figure 5B). In line with this result, epididymal *Glut4* expression was significantly decreased in both leptin-treated rats (Figure 5B). These data agree with the fact that subcutaneous FGF21 treatment increase pERK1/2 levels as a consequence of the increase in circulating levels of FGF21, as previously reported [42]. All these results might suggest that eWAT response/sensitivity to circulating FGF21 is potentiated in leptin-treated rats and point, for the first time, to a clear role of PPARβ/δ and central leptin in modulation of FGF receptors expression and FGFR1-β-klotho receptor complex levels in eWAT, which, according with recent reports [43,44], affect the sensitivity of eWAT to FGF21.

### 2.5. PPARβ/δ Activity Is Essential in Promotion of FGF21 Endogenous Expression and Browning Program in eWAT Induced by Central Leptin

It is known that endocrine FGF21 action is largely due to liver-secreted FGF21. However, under certain conditions adipocytes increase their own production of FGF21. The adipose tissue-derived FGF21 is thought to act in an autocrine/paracrine manner on adipocytes, independently of serum levels of the hormone. In this regard it has been demonstrated that male Wistar rats acclimated to cold (4 °C for 7 days) reduced their circulating levels of FGF21 but increased FGF21 secretion from white adipocytes [22,45]. Then, we analyzed mRNA and protein levels of FGF21 in eWAT.

In non-leptin-treated rats, GSK0660 treatment did not modify mRNA nor protein levels of adipose FGF21 in eWAT (Figure 5C,D). However, leptin administration acutely increased *Fgf21* gene expression in eWAT and potentiate protein levels by ~3-fold when compared with both vehicle-infused rats (SS and PF) (Figure 5E,F). Interestingly, inhibition of PPARβ/δ activity during leptin infusion practically abolished FGF21 abundance in eWAT (Figure 5F). These results might suggest that indirect induction of PPARβ/δ by central leptin is implicated in the control of FGF21 production by eWAT.

Lastly and because previous studies demonstrated that adipose tissue ‘browning’ have been observed in response to FGF21 administration [21,46], we analyzed whether brain leptin-PPARβ/δ pathway could play a role in the transcriptional program that control white fat metabolic reprogramming during adrenergic stimulation. First, we tested β3-adrenergic receptor expression and expression levels of several markers of browning, as well as protein levels of the main uncoupling protein UCP-1 and the content of mitochondrial oxidative phosphorylation complex (OXPHOS) in eWAT, to elucidate the role of central leptin and the effect of PPARβ/δ inhibition on the transcriptional regulation of the browning process.

**Figure 6:**
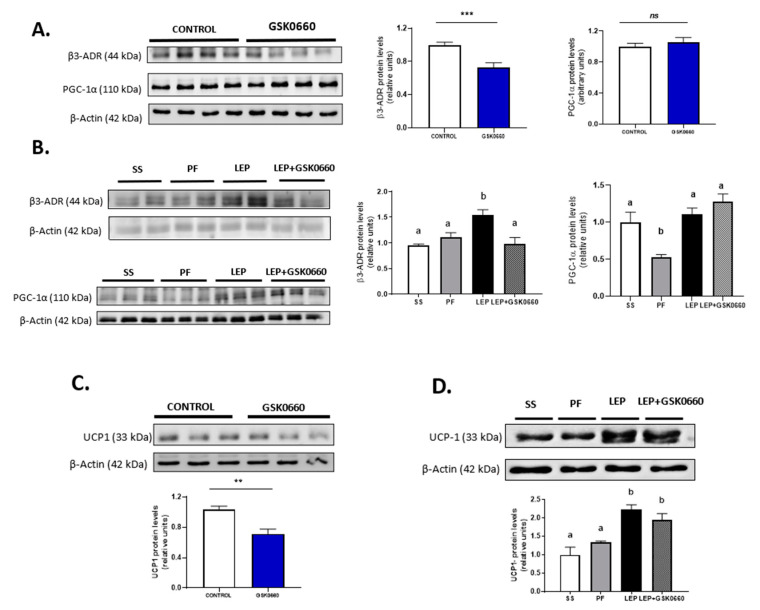
Representative Western blot of β3-adrenergic receptor, PGC-1α (**A**) and UCP-1 (**C**) protein levels and relative densitometric analysis in eWAT of rats after 7 days of vehicle (DMSO) control or intraperitoneal antagonist GSK0660 injection (*n* = 6). (**B**) Representative Western blot of β3-adrenergic receptor, PGC-1α (**B**) and UCP-1 (**D**) protein levels and relative densitometric analysis in eWAT of rats after 7 days of chronic central treatment of vehicle (SS and PF), leptin, or leptin and ip GSK0660 co-administration (*n* = 4–6). Densitometric levels of proteins are expressed relative to the Control group and SS group, the levels of which were set at 1.0 arbitrarily. Results are the mean ± SEM per group of animals (*n* = 4–6). Differences between CONTROL vs. GSK0660 treatment were analyzed by Student’s *t*-test (** *p* < 0.05, *** *p* < 0.001; ns: non-significant). Significant differences between ICV-treated rats were analyzed by One-Way ANOVA followed by Tukey test (differences letters mean significant differences among treatments, *p* ≤ 0.05). ^a^ *p* ≤ 0.05 vs. PF or Lep or Lep+GSK0660; ^b^ *p* ≤ 0.05 vs. SS or PF or Lep+GSK0660. SS: vehicle-infused rats fed ad libitum, PF: vehicle-infused pair-fed rats; Lep: leptin-infused rats; Lep+GSK0660: leptin-infused rats plus PPARβ/δ antagonist GSK0660.

The administration of GSK0660 notably decreased both *β3-Adr* and *Pparɣ* gene expression in non-leptin-treated rats (Table 3), being the latter one of the inductors of browning and *Fgf21* expression in WAT. The protein levels of β3-adrenergic receptor were also reduced in eWAT after inhibiting PPARβ/δ activity (Figure 6A). In contrast, the gene expression of PPARɣ co-activator, *Pgc-1α*, was increased by GSK0660 treatment although its protein abundance was similar to the control levels (Table 3, Figure 6A). As expected, total UCP-1 protein levels were markedly reduced in eWAT after GSK0660 administration (Figure 6C) as well as the gene expression of the browning marker *P2rx5*, remaining *Prdm16* and *Tbx15* mRNA levels unchanged (Table 3).

Leptin infusion induced β3-Adr mRNA and protein levels in eWAT (Table 3, Figure 6B). Moreover, inhibition of PPARβ/δ upon leptin administration decreased the mRNA levels of both *β3-Adr* and *Pparɣ*, and β3-adrenergic receptor protein levels (Table 3, Figure 6B). In addition, mRNA levels of *Pgc-1α* were notably increased when icv leptin and ip GSK0660 were co-administered, without overtly changes in its protein levels (Table 3, Figure 6B). Interestingly, chronic icv leptin administration was able to increase UCP-1 protein levels in eWAT and partially prevented the decrease of this protein induced by the pharmacological inhibition of PPARβ/δ (Figure 6D). Furthermore, leptin administration also increased the expression of the browning markers *Prdm16* and *P2rx5*, and GSK0660 notably downregulated both genes when was co-administered with leptin (Table 3), indicating that GSK0660 decreases the browning capacity of eWAT after central leptin infusion.

It has been reported that in *ob/ob* mice, 30% of the weight loss induced by the subcutaneous injection of leptin is due to changes in energy expenditure [13]. Moreover, these authors described that in *ob/ob* mice leptin decreased the protein levels of three substrate oxidation complexes (OXPHOS) in liver and heart, following by a reduction in basal metabolic rate and mitochondrial volume density. In agreement with these data, we have recently reported similar results in heart after chronic icv leptin infusion in rats [28]. Hence, we investigate whether leptin and/or the pharmacological inhibition of PPARβ/δ could alter mitochondrial activity driven by OXPHOS complexes in eWAT. In addition, we measured the mRNA levels of the transcription factor NF-E2-related factor 2 (*Nrf2*) that plays a key role in the maintenance of cellular redox balance and detoxification [47].

**Figure 7:**
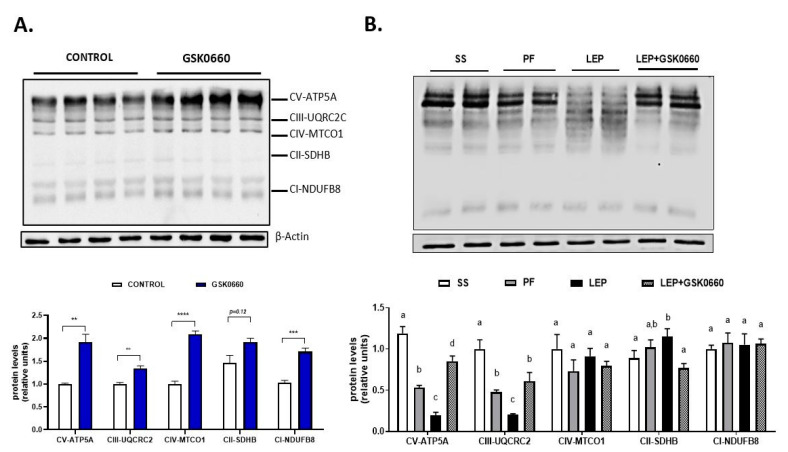
(**A**) Representative Western Blot of OXPHOS complexes 1–5 and relative densitometric analysis in eWAT of rats after 7 days of vehicle (DMSO) control or intraperitoneal antagonist GSK0660 injection (*n* = 6). (**B**) Representative Western blot of OXPHOS complexes 1–5 and relative densitometric analysis in eWAT of rats after 7 days of chronic central treatment of vehicle (SS and PF), leptin, or leptin and ip GSK0660 co-administration (*n* = 4–6). Densitometric levels of proteins are expressed relative to the Control group and SS group, the levels of which were set at 1.0 arbitrarily. Results are the mean ± SEM per group of animals (*n* = 4–6). Differences between CONTROL vs. GSK0660 treatment were analyzed by Student’s *t*-test (** *p* <0.1, *** *p* < 0.001, **** *p* < 0.0001). Significant differences between ICV-treated rats were analyzed by One-Way ANOVA followed by Tukey test (differences letters mean significant differences among treatments, *p* ≤ 0.05). ^a^ *p* ≤ 0.05 vs. PF or Lep or Lep+GSK0660; ^b^ *p* ≤ 0.05 vs. SS or Lep or Lep+GSK0660; ^c^ *p* ≤ 0.05 vs. SS or PF or Lep+GSK0660. SS: vehicle-infused rats fed ad libitum, PF: vehicle-infused pair-fed rats; Lep: leptin-infused rats; Lep+GSK0660: leptin-infused rats plus PPARβ/δ antagonist GSK0660.

In non-leptin-treated rats, *Nrf2* gene expression in eWAT was notably decreased upon antagonist GSK0660 administration (Table 4). Nevertheless, GSK0660 increased ~2-fold the abundance of OXPHOS complexes CV, CIII, CIV, and CI (Figure 7A). Hence, these data suggest that PPARβ/δ is involved in the control of mitochondrial OXPHOS complexes content in eWAT. On the other hand, chronic leptin infusion notable increased *Nrf2* mRNA levels when compared with pair-fed rats, but this effect was abolished when GSK0660 was co-administered with leptin (Table 4). Two subunits of OXPHOS system, complexes V and III, the main site of ATP synthesis and ROS production, respectively, were significantly decreased by central leptin treatment compared with pair-fed control rats (Figure 7B). Curiously, low levels of ATP-synthase are considered as an important feature of rodent brown adipose tissue and have also described in brite-fat mitochondria [48,49]. Interestingly, PPARβ/δ inhibition abolished the effects of leptin in mitochondrial OXPHOS complexes content in eWAT (Figure 7B).

## 3. Discussion

In the current study, we analyze the implication of PPARβ/δ as a potential mediator of both central and peripheral metabolic, inflammatory, and immunomodulatory actions of leptin acting on the CNS. Because currently little is known about the role of PPARβ/δ in visceral WAT, we focus our attention on the in vivo blockage of PPARβ/δ in eWAT from chronic icv leptin treated rats. Herein, we demonstrate that leptin, acting through hypothalamic neural circuits and the SNS, increases PPARβ/δ expression in eWAT and that leptin effects on browning and inflammation of visceral adipose tissue are partially regulated by PPARβ/δ. Moreover, our results also unveil that PPARβ/δ is involved in the up-regulation of FGF21 expression induced by leptin in eWAT.

Neuronal-specific deletion of PPARβ/δ has been reported to lead to general leptin insensitivity after treatment with ip leptin [32]. In this sense, an important finding of our study is that pharmacological inhibition of PPARβ/δ generates insensitivity to the appetite-suppressing effects and therefore to the anti-lipidemic effects of brain leptin in eWAT.

PPARβ/δ is a sensor of the cellular lipid environment and a regulator of inflammation and oxidative stress [26]. As a receptor, it binds lipid molecules that activate its ability to modify gene expression and then the behavior of cells in a new environment. Overexpression of a constitutive active form of PPARβ/δ in Lepr^db/db^ mice adipose tissue decreased adiposity and reversed the obesity phenotype [25]. In support of this, these authors showed that an acute treatment with a PPARβ/δ agonist GW501516 depletes WAT lipid accumulation in these genetically obese mice, suggesting that the anti-obesity actions of PPARβ/δ are not dependent on leptin signaling pathways [25]. Nevertheless, PPARβ/δ-null mice but not adipose tissue-specific PPARβ/δ-knockout mice are extremely lean and have reduced adipose stores [50,51]. Furthermore, several lines of evidence suggest that PPARβ/δ and leptin signaling are interconnected, because neuronal-specific PPARβ/δ knockout mice are leptin resistant [32] and downregulation of leptin upon PPARβ/δ activation have been seen in liposarcoma cells [52].

In the present study we provide new insights on the mechanisms by which chronic central leptin administration exert its anti-obesity actions through the CNS. These involve the activation of PPARβ/δ and the improvement of the browning capacity in eWAT. In this regard, our data show that leptin acting through the SNS dampens the effects of GSK0660 on the expression of leptin in eWAT. Secondly, the pharmacological inhibition of PPARβ/δ upon ip GSK0660 injection, a selective antagonist inhibitor of PPARβ/δ, abolishes the effects of icv leptin on the expression of *Ob-Rb* in eWAT, which could impair the direct effect of leptin on eWAT metabolism and innate/adaptive immune response.

In fact, we found that GSK0660 treatment appears to change the immune cells balance in eWAT towards a more inflammatory profile, but flow cytometry analysis is needed to validate these observations. In addition, inhibition of PPARβ/δ increases *Npy* but decreases *Ccl5* expression in hypothalamus from icv leptin treated rats, which correlates with the abolishment of the anorexigenic effects of leptin by GSK0660. Thus, our data support that PPARβ/δ could be involved in the crosstalk between CNS, immune system, and the remodeling of visceral WAT promoted by the hormone/cytokine leptin.

On the other hand, our data shed a new light on the own PPARβ/δ metabolic action on eWAT. In the absence of ligands and/or ligand precursors generated by increased lipolysis in eWAT in response to brain leptin infusion [6,7], the pharmacological inhibition of PPARβ/δ decreased *PPARγ* expression and increases cholesterol and TAG content in eWAT and, consequently, the mRNA levels of both *leptin* and the main leptin receptor *Ob-Rb.* More importantly, GSK0660 administration markedly decreased the endogenous FGF21 content in eWAT and increased the levels of OXPHOS system in this tissue. Lower expression of *PPARγ* is linked to the pathogenesis of metabolic syndrome [53], whereas increased OXPHOS have been associated to increased basal respiration and oxidative stress [54,55] a characteristic of adipocytes from insulin resistant obese-nondiabetic humans and mice [56]. In our setting, GSK0660 alone increases circulating levels of insulin an indicator of insulin resistance.

FGF21 plays very important roles in the regulation of energy dissipation and balance [16,17,57]. Here, we report for the first time that chronic leptin administration notably decreased the circulating levels of FGF21, independently of the anorexigenic properties of leptin and/or the presence of GSK0660. In parallel, central leptin infusion increased the components of FGF21 signaling machinery in eWAT compared with pair-fed rats, supporting the idea that leptin through the brain controls the visceral adipose tissue response to FGF21. Although there is little evidence on the relationship between FGF21 signaling and PPARβ/δ activity in visceral WAT, our results suggest that global inhibition of PPARβ/δ could reduce the potential endocrine action of FGF21 in eWAT.

In addition, icv leptin infusion increased the expression of several browning hallmark markers as *Pdk4, β3-Adr, Prdm16*, and *P2rx5*, as well as β3-Adr, PGC-1α, and UCP1 protein levels in eWAT. These results indicate the presence of cells within this visceral WAT depot with a gene expression pattern similar to “beige” or “brite” cells [58,59] confirming leptin’s browning capacity in eWAT. Interestingly, these results correlate with the acutely increase of FGF21 expression in eWAT by leptin infusion.

Previous studies have described PPARβ/δ as a candidate for browning induction in fat cells [25,60,61]. In line with these observations, the pharmacological inhibition of PPARβ/δ by GSK0660 decreased *Pdk4, β3-Adr*, *PPARɣ,* and *P2rx5* gene expression and β3-Adr and UCP-1 protein levels in eWAT from rats chronically treated with icv leptin, confirming that leptin action on the CNS and via SNS activates lipolysis [6,7] generating tissue-specific PPARβ/δ ligands and/or ligand precursors which, once present in adipose tissue, might also induce the browning capacity of this tissue. Nevertheless, further work has to be done in order to confirm the molecular nature of the ligands that might activate PPARβ/δ in eWAT, in response to the increased lipolysis induced by central leptin in this tissue.

**Figure 8:**
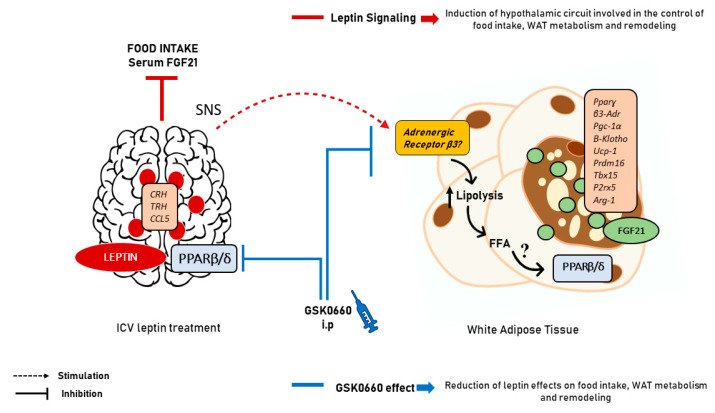
Scheme summarizing the effects of central leptin on eWAT. Leptin, acting through hypothalamic neural circuits and the SNS activates lipolysis in eWAT, generating probably tissue-specific PARβ/δ ligands and/or ligand precursors which, once present in adipose tissue might activate PPARβ/δ function in eWAT inducing the endogenous expression of FGF21 and the browning capacity of this tissue. The molecular nature of the ligands that might activate PPARβ/δ in eWAT, in response to the increased lipolysis induced by central leptin in this tissue is not known and remains to be determined. SNS: sympathetic nervous system; FFA: free fatty acids.

It was unexpected that the pharmacological inhibition of PPARβ/δ with the specific antagonist GSK0660 totally blunted the endogenous FGF21 protein content of eWAT in leptin treated rats. Although FGF21 expression and effects, including its browning capacity, are closely associated with PPARα and PPARɣ activity [62,63], a study in humans with PPARs agonists revealed that treatment with the PPARβ/δ agonist (GW501516) increased the circulating levels of FGF21, providing evidence of a metabolic relationship between PPARβ/δ isoform and FGF21 expression [64]. Our results suggest that the activation of PPARβ/δ in visceral adipose tissue in response to icv leptin infusion regulates the endogenous expression of FGF21 contributing to white-to-beige cell transition in eWAT (summarized in Figure 8). In this sense, direct central leptin action in the hypothalamus, and/or indirect effects mediated by SNS on eWAT and heart [28], could increase energy expenditure and decrease body weight in rats.

Finally, it has been generally stated that impaired mitochondrial phosphorylation becomes a metabolic disturbance underlying insulin resistance and obesity development in humans and rodents [55]. Nevertheless, other works revealed that impaired OXPHOS function can increase lifespan in *Drosophila melanogaster, Caenorhabditis elegans,* and rodents [65,66,67]. In addition, genetic models characterized by decreased adipocyte OXPHOS point to a protective role of FGF21 in mediating tissue specific adaptation to this cellular stress [14,68]. Although the specific mechanism by which the production of FGF21 by visceral adipose tissue has not been described, herein we show that PPARβ/δ could be an essential factor for inducing endogenous expression of FGF21 in eWAT in response to central leptin signaling. Furthermore, the pharmacological inhibition of PPARβ/δ by the antagonist GSK0660 has a huge impact in visceral fat accretion, reduction of FGF21 endogenous production and white-to-beige fat transformation in eWAT. This study has some limitations. Despite the lipophilic nature of GSK0660, we do not have direct confirmation on whether the intraperitoneal GSK0660 administration can pass the brain–blood barrier (BBB) leading to the inhibition of the hypothalamic PPARβ/δ activation mediated by central leptin. However, several lines of evidences reported herein support that GSK0660 can indeed pass the BBB. The pharmacological inhibition of PPARβ/δ increased *Npy* and decreased *Ccl5* expression in the hypothalamus, both involved in the hypothalamic control of food intake and regulated by central leptin in an opposite way. On the other hand, GSK0660 administration blunted the anorectic and adipostatic effects induced by leptin in the brain leading to visceral fat accretion and reducing both the endogenous FGF21 protein content and the browning capacity of this tissue. However, we do not know whether the regulation of CCL5 expression by leptin in hypothalamus requires signals from the periphery or is linked to a central local effect, or whether the effect of GSK0660 on hypothalamic neurons is direct or indirect. Hence, further work has to be done before a final conclusion can be drawn about this issue. Overall, our results contribute to understand the mechanisms by which PPARβ/δ and leptin acting through the CNS exert its effects on eWAT. Nevertheless, future studies are needed to determine how leptin enhances central or peripheral PPARβ/δ activity and to test the role of PPARβ/δ as a potential mediator of leptin in the brain–visceral fat crosstalk.

## 4. Materials and Methods

### 4.1. Experimental Animals

Experiments were performed in male 3-month-old Wistar rats. Animals were randomly housed individually, to control their food intake and thereby avoid differences in adipose tissue weight and serum levels of hormones and metabolites that depend on the amount of feed eaten by animals. All the animals were fed with a standard chow diet and water and maintained in ventilated-controlled quarters (20–25 °C temperature, 50–55% humidity, and 12 h-light cycle 8 a.m.–8 p.m.). Body weight and food intake was monitored daily during all the treatments. Animals were handled according to European Union’s laws (2010/63/EU) and following Spanish regulations (RD 53/2013) for laboratory animal use. Experimental protocols with animals were approved by the Institutional Committee for Ethical Animal Care CEEA/UCLM (Permit Numbers CE/301012 and CE/99-1835-A308, research proposal approved respectively, on 30 October 2012 and 17 May 2019). All efforts were made to reduce the number of animals used and minimize animal suffering.

### 4.2. PPARβ/δ Antagonist (GSK0660) Administration

Pharmacological antagonist GSK0660 for PPARβ/δ inhibition was administered in both experiments as previously described at a dose that does not induce toxic side effects in rats [69,70]. Briefly, GSK0660 was diluted first in DMSO (less than 1%) and later in 0.9% NaCl. Then, it was injected daily ip (1 mg/kg per day) during 7 days to GSK0660 group and LEP+GSK0660 group of rats, while the CONTROL and LEP groups received in parallel an ip injection of vehicle at 2 mL/kg (0.062% DMSO). After 7 days of treatment, all animals were fasted (overnight 12 h), anesthetized by CO_2_ inhalation and sacrificed by decapitation at 09:30 a.m. Tissue samples were rapidly dissected and snap frozen in liquid nitrogen. Serum was obtained by centrifugation (1000 *g*, 10 min).

### 4.3. Intracerebroventricular Leptin Administration

The ICV leptin (LEP, LEP+GSK0660) or vehicle administration (SS, PF) in young 3-month-old rats was performed as described [27,28] (see Supplementary information for details). To evaluate the role of PPARβ/δ on central leptin effects, the group LEP+GSK0660 was infused with leptin and co-administered ip with antagonist GSK0660 as previously described. Body weight and food intake was measured daily during the experiment. After 7 days of experiment, animals were sacrificed as previously described (see “PPARβ/δ antagonist GSK0660 administration” section).

### 4.4. Biochemical Assessment

Serum hormone and metabolites levels were measured as described [27,28] (see Supplementary information for details). Triglyceride and cholesterol content in epididymal white adipose tissue was extracted from 100 mg frozen tissue by chloroform-methanol (2:1 *v/v*) and was determined using enzymatic reagents following manufacturer’s instructions (Triglyceride: #11528; cholesterol: #11505; BioSystems, Barcelona, Spain).

### 4.5. Quantitative Transcription Analysis with Real Time Polymerase Chain Reaction (qRT-PCR)

Total RNA was isolated from frozen tissues using RNeasy Lipid Tissue kit following manufacturer’s instructions (#74804, Qiagen, Venlo, Netherlands) after homogenization with 1 mL of Qiazol (#79306, Qiagen). Gene expression was performed by using ABI PRISM 7500 Fast Sequence Detection System instrument and software (Applied Biosystem, Foster City, CA, USA). Relative quantification of target cDNA in each sample was performed from cDNA in TaqMan One-Step real time PCR Master Mix (#4352042, PE Applied Byosystems, Foster City, CA, USA), using Pre-Developed TaqMan Assay Reagents (PE Applied Biosystem) for *β-Klotho*, *Ccl5*, *Crh*, *Cpt-1α*, *Fgf21*, *Fgfr1*, *Fgfr2*, *Fgfr3*, *Fgfr4*, *Glut4*, *Nrf2*, *Pgc-1α*, *P2rx*, *5 Prdm16*, *Pparα*, *Pparβ/δ*, *Pparɣ,* and *Tbx15* with FAM and 18S rRNA with VIC as real time reporter, according to manufacturer’s protocol, whereas *Npy*, *Pomc*, *Trh*, *ObRb*, and *Leptin (ob)* we used SYBR-Green One-Step real time PCR Master Mix was used (#4309156, PE Applied Byosystems, Foster City, USA) according to manufacturer instructions with primers supplied by Sigma-Aldrich (see Supplemental Tables S1 and S2 for details).

### 4.6. Epididymal White Adipose Tissue (eWAT) Total Extract Preparation

Tissues (100 mg) were homogenized in a manual Dounce homogenizer and lysed in homogenization buffer (50 mM Hepes-KOH, pH 7.4; 150 mM NaCl, 2 mM EDTA, 0,5% deoxycolate) containing proteases and phosphatases inhibitors (1 mM PMSF, 2 mM NaF, 10 µg/mL leupeptin, 10 µg/mL pepstatin, 10 µg/mL aprotinin, and 2 mM Na3VO4). The homogenate was centrifugated 10,000 rpm for 10 min at 4 °C to produce a total protein lysate of eWAT. All extracts were stored at −80 °C until use. Bradford protein assay was used for total protein quantification (#500-006, Bio-Rad, Hercules, CA, USA).

### 4.7. Hypothalamus Total RNA and Protein Extract Preparation

The hypothalamic regions were carefully dissected as previously described [71]. After that, hypothalamic regions were frozen in liquid nitrogen and stored at −80 °C until further processing. RNA and protein from hypothalamus were obtained using All Prep DNA/RNA/Protein Mini kit (Cat. No. 80004, Qiagen, Venlo, The Netherlands) following manufacturer’s instructions. The cDNA was synthesized from 1.5 μg of DNase-treated RNA. Bradford protein assay was used for total protein quantification (#500-006, Bio-Rad, CA, USA).

### 4.8. Western Blot Analysis and Inmunoblotting

Protein lysates (equal amounts of 20–30 µg) were separated under reducing condition (7.5–10% polyacrylamide concentration gels) SDS-PAGE. Samples were previously mixed with SDS sample buffer and boiled at 95 °C for 10 min. Proteins were transferred to nitrocellulose sheets (0.2 µm, Bio-Rad) and incubate overnight (12–16 h) at 4 °C with the appropriate primary antibodies, followed by incubation at room temperature for 2 h with corresponding secondary antibody conjugated with horseradish peroxidase (see Supplemental information for further details).

### 4.9. Statistical Analysis

Data are expressed as mean ± SEM. Statistical analysis was performed using the GraphPad Prism version 8.4 for Windows (GraphPad Software, San Diego, CA, USA). Differences between two groups were assessed using the unpaired Student’s *t*-test. Significant differences between more than two groups were assessed by one-way ANOVA followed by Tukey test as post-hoc analysis (different letters indicate significant differences among treatments). All differences were considered significant at *p* < 0.05. The number of rats used per experiments is stated in each figure legend.

## Figures and Tables

**Table 1:** Effects of GSK0660 and ICV leptin administration on serum metabolites and hormone levels of the animals.

TREATMENT	CONTROL	GSK0660	SS	PF	LEP	LEP+GSK0660
Glucose (mM)	6.95 ± 1.06	6.58 ± 1.69	4.37 ± 0.37 ^a^	4.52 ± 0.62 ^a^	4.75 ± 1.7 ^a^	4.18 ± 0.4 ^a^
Insulin (ng/mL)	1.32 ± 0.12	2.51 ± 0.72 **	1.6 ± 0.5 ^a^	1.4 ± 0.1 ^a^	0.7 ± 0.1 ^b^	0.63 ± 0.19 ^b^
Cholesterol (mg/dL)	76.83 ± 14.29	86.87 ± 3.09	76.67 ± 9.9 ^a^	51.46 ± 5.2 ^b^	52.4 ± 18 ^b^	60.8 ± 11.7 ^a,b^
Triglycerides (mg/dL)	132.74 ± 13.27	177.88 ± 19.47 *	85 ± 7 ^a^	80 ± 5 ^a^	53 ± 6 ^b^	90± 15 ^a^
Leptin (ng/mL)	4.01 ± 0.92	9.01 ± 0.26 *	6.01 ± 0.5	5.4 ± 1	5.2 ± 0.8	7.16 ± 1.6
FGF21 (pg/mL)	266.54 ± 21.25	251.14 ± 31.10	20 ± 4.44 ^a^	129.92 ± 5.2 ^b^	77.11 ± 11.09 ^c^	60.38 ± 14.05 ^c^

After 7 days of GSK0660 intraperitoneal and/or central leptin (LEP, 0.2 µg/day) or vehicle (SS, PF) administration, serum, and metabolites hormone levels. Results are represented by mean ± SEM (n = 6) per group of animals. Differences between CONTROL vs. GSK0660 treatment were analyzed by Student’s *t*-test (* *p* < 0.05, ** *p* < 0.001). Significant differences between ICV-treated rats were analyzed by One-Way ANOVA followed by Tukey test (differences letters mean significant differences among treatments, *p* ≤ 0.05). ^a^ *p* ≤ 0.05 vs. PF or Lep or Lep+GSK0660; ^b^ *p* ≤ 0.05 vs. SS or PF or Lep+GSK0660; ^c^ *p* ≤ 0.05 vs. SS or PF. SS: vehicle-infused rats fed ad libitum; PF: vehicle-infused pair feed rats; LEP: leptin-infused rats; Lep+GSK0660: leptin-infused rats plus PPARβ/δ antagonist GSK0660.

**Table 2:** Gene expression of *leptin* and *ObRb* leptin receptor in epididymal white adipose tissue.

Gene	CONTROL	GSK0660	SS	PF	LEP	LEP+GSK0660
*Leptin*	1.05 ± 0.12	2.18 ± 0.17 **	1.02 ± 0.08 ^a^	2.37 ± 0.22 ^b^	0.27 ± 0.05 ^c^	0.32 ± 0.14 ^c^
*ObRb*	1.07 ± 0.28	3.1 ± 0.35 **	0.98 ± 0.18 ^a^	3.3 ± 0.37 ^b^	2.36 ± 0.6 ^b^	0.06 ± 0.018 ^c^

Results are represented by mean ± SEM (n = 6) per group of animals. Data from real-time RT-PCR are expressed relative to 18S rRNA and the mRNA levels are expressed relative to the Control group and SS group the level of which were set at 1.0 arbitrarily Differences between CONTROL vs. GSK0660 treatment were analyzed by Student’s *t*-test (** *p* < 0.001). Significant differences between ICV-treated rats were analyzed by One-Way ANOVA followed by Tukey test (differences letters mean significant differences among treatments, *p* ≤ 0.05). ^a^ *p* ≤ 0.05 vs. PF or Lep or Lep+GSK0660; ^b^ *p* ≤ 0.05 vs. SS or PF or Lep+GSK0660; ^c^ *p* ≤ 0.05 vs. SS or PF or Lep. SS: vehicle-infused rats fed ad libitum; PF: vehicle-infused pair feed rats; LEP: leptin-infused rats; Lep+GSK0660: leptin-infused rats plus PPARβ/δ antagonist GSK0660.

**Table 3:** Gene expression of *β3-Adr, Pparɣ, Pgc-1a*, *Prdm16, Tbx15*, and *P2rx5* in eWAT.

Gene	CONTROL	GSK0660	SS	PF	LEP	LEP+GSK0660
*β3-Adr*	1.10 ± 0.13	0.66 ± 0.06 **	1.06 ± 0.06 ^a^	1.25 ± 0.2 ^a^	1.83 ± 0.12 ^b^	0.61 ± 0.05 ^c^
*Pparɣ*	0.97 ± 0.22	0.56 ± 0.01 *	1.10 ± 0.04 ^a^	1.10 ± 0.02 ^a^	1.05 ± 0.013 ^a^	0.65 ± 0.06 ^b^
*Pgc-1a*	1.02 ± 0.2	1.43 ± 0.15 *	0.98 ± 0.25 ^a^	1.2 ± 0.11 ^a^	0.99 ± 0.23 ^a^	4.64 ± 0.48 ^b^
*Prdm16*	1.1 ± 0.27	1.3 ± 0.15	1.04 ± 0.06 ^a^	1.06 ± 0.25 ^a^	1.82 ± 0.02 ^b^	1.4 ± 0.3 ^a^
*Tbx15*	0.97 ± 0.45	1.2 ± 0.47	0.89 ± 0.18 ^a^	2.09 ± 0.6 ^b^	1.58 ± 0.3 ^b^	1.1 ± 0.48 ^a^
*P2rx5*	1.14 ± 0.06	0.7 ± 0.01 **	1.06 ± 0.13 ^a^	1.15 ± 0.16 ^a^	3.13 ± 0.38 ^b^	1.25 ± 0.19 ^a^

Results are represented by mean ± SEM (*n* = 6) per group of animals. Data from real-time RT-PCR are expressed relative to 18S rRNA and the mRNA levels are expressed relative to the Control group and SS group, the level of which was set at 1.0 arbitrarily. Differences between CONTROL vs. GSK0660 treatment were analyzed by Student’s *t*-test (* *p* < 0.05, ** *p* < 0.001). Significant differences between ICV-treated rats were analyzed by One-Way ANOVA followed by Tukey test (differences letters mean significant differences among treatments, *p* ≤ 0.05). ^a^ *p* ≤ 0.05 vs. PF or Lep or Lep+GSK0660; ^b^ *p* ≤ 0.05 vs. SS or PF or Lep+GSK0660; ^c^ *p* ≤ 0.05 vs. SS or PF or Lep. SS: vehicle-infused rats fed ad libitum, PF: vehicle-infused pair-fed rats; Lep: leptin-infused rats; Lep+GSK0660: leptin-infused rats plus PPARβ/δ antagonist GSK0660.

**Table 4:** Gene expression of *Nrf2* in epididymal white adipose tissue.

Gene	CONTROL	GSK0660	SS	PF	LEP	LEP+GSK0660
*Nrf2*	1.10 ± 0.07	0.73 ± 0.02 **	1.07 ± 0.07 ^a^	0.86 ± 0.06 ^a^	1.88 ± 0.08 ^b^	0.97 ± 0.045 ^a^

Results are represented by mean ± SEM (*n* = 6) per group of animals. Data from real-time RT-PCR are expressed relative to 18S rRNA and the mRNA levels are expressed relative to the Control group and SS group, the level of which were set at 1.0 arbitrarily Differences between CONTROL vs. GSK0660 treatment were analyzed by Student’s *t*-test (** *p* < 0.001). Significant differences between ICV-treated rats were analyzed by One-Way ANOVA followed by Tukey test (differences letters mean significant differences among treatments, *p* ≤ 0.05). ^a^ *p* ≤ 0.05 vs. Lep; ^b^ *p* ≤ 0.05 vs. SS or PF or Lep+GSK0660. SS: vehicle-infused rats fed ad libitum, PF: vehicle-infused pair-fed rats; Lep: leptin-infused rats; Lep+GSK0660: leptin-infused rats plus PPARβ/δ antagonist GSK0660.

## Data Availability

Al relevant data are included within the manuscript and in the Supplementary Materials.

## Supplementary Materials

The following are available online at https://www.mdpi.com/article/10.3390/ijms22094624/s1, Figure S1: FGF21 mRNA and protein levels in liver of rats after 7 days of chronic central treatment; Table S1: Probes used for real time PCR, Table S2: primers sequences used for real time PCR; Table S3: mRNA levels of Ob-Rb, Pomc, Crh and Trh in hypothalamus.

## Abbreviations

ARC	arcuate nucleus
Arg1	Arginase-1
ATGL	Adipose triglyceride lipase
Ccl5	chemokine ligand 5
CNS	central nervous system
CPT-1a	Carnitine Palmitoyltransferase 1a
Crh	corticotropin releasing hormone
ERK1/2	extracellular signal-regulated kinase 1/2
FGF21	Fibroblast Growth Factor-21
FGFR	FGF21 receptor
GLUT4	Glucose transporter 4
HPT	hypothalamic–pituitary–thyroid axis
HSL	hormone-sensitive lipase
Nrf-2	nuclear factor erythroid 2-related factor 2
Npy	neuropeptide Y
ObRb	Long form of the leptin receptor
OXPHOS	Mitochondrial oxidative phosphorylation system
Pdk4	Pyruvate Dehydrogenase Kinase 4
P2rx5	purinergic receptor P2X 5
Pgc-1α	Peroxisome proliferator-activated receptor gamma coactivator 1α
POMC	proopiomelanocortin neurons
PPARα	Peroxisome proliferator-activated receptor-α
PPARβ/δ	Peroxisome proliferator-activated receptor-β/δ
PPARγ	Peroxisome proliferator-activated receptor-γ
Prdm16	Pr-domain containing protein 16
SNS	sympathetic nervous system
Tbx15	Transcription factor 15
Trh	thyrotropin-releasing hormone
UCP-1	Uncoupling protein-1
