# Supplementary material for: Leptin, Acting at Central Level, Increases FGF21 Expression in White Adipose Tissue via PPARβ/δ
Source: Int J Mol Sci. 2021 Apr 28;22(9):4624. doi: 10.3390/ijms22094624 (PMC8124190; doi:10.3390/ijms22094624)
Supplement: Supplementary file 1 [file ijms-22-04624-s001.zip › ijms-1190288-supplementary.pdf]

Supplemental Methodology

Intracerebroventricular leptin administration

Rats were anesthetized by inhalation a mixture of O2 and isoflurane (Pharmacia-Upjohn, Barcelona, Spain), and then, were placed in a stereotaxic frame (David Kopf, Tujunga California, USA) with a thermal blanket below. Leptin or saline (PBS) was administered during 7 days in the lateral ventricle through a cannula connected to an osmotic minipump (Alzet, Palo Alto, CA), with a releasing rate of 1 µl/h, and filled with 0.0082 µg/µl (0.2 µg/day) rat leptin (Sigma), or its vehicle (saline). Rats, (n = 6 per group) were treated with either saline (SS) or leptin (Lep) for 7 days. A third group of rats (PF) was treated with saline and pair-fed to the amount of food consumed by the leptin-treated group.

Biochemical Assessment

Blood was removed and centrifuged (2000 xg, 15 min), serum was recovered and frozen in liquid nitrogen for stored at –70ºC until use. Serum hormone and metabolite levels were measured following manufaturer's protocol: glucose (Accutrend Glucose Analyser, #05050472223, Roche); insulin (Rat Insulin Elisa #10-1251-01 Mercodia, Sweden); leptin (Rat Elisa Kit #RD291001200R, Biovendor, Germany), tryglicerides (#11528, Biosystems, Barcelona, Spain), cholesterol (#11505, Biosystems, Barcelona, Spain), FGF21 (FGF21 mouse/rat Elisa #RD291108200R, BioVendor, Germany).

Quantitative transcription analysis with real time polymerase chain reaction

The cDNA was synthesized from 1.5 µg of DNase-treated RNA. Relative quantification of Arginase-1 (*Arg-1*), β-klotho co-receptor (*β-klotho*), chemokine ligand 5 (*Ccl5*), corticotropin releasing hormone (*Crh*), Carnitine Palmitoyltransferase 1A (*Cpt-1a*), fibroblast growth factor 21 (*Fgf21*), fibroblast growth factor receptors 1-4 (*Fgfr1, Fgfr2, Fgfr3, Fgfr4*), glucose transporter 4 (*Glut4*), nuclear factor erythroid 2-related factor 2

(*Nrf2*), neuropeptide Y (*Npy*), peroxisome proliferator-activated receptor gamma co-activator 1-alpha (*Pgc-1a*), purinergic receptor P2X 5 (*P2rx5*), PR domaing containing 16 (*Prdm16*), peroxisome proliferator-activated receptor γ (*Pparγ*), peroxisome proliferator-activated receptor β/δ (*Pparβ/δ*), peroxisome proliferator-activated receptor α (*Pparα*), proopiomelanocortin (*Pomc*), T-box Transcription factor 15 (*Tbx15*) and Uncoupling protein 1 (*Ucp-1*) mRNA levels was performed by real-time PCR according to the manufacturer's protocol on an ABI PRISM 7500 FAST Sequence Detection System instrument and software (PE Applied Biosystem, Foster City, CA). To standardize the amount of cDNA added to the reaction, amplification of endogenous control 18S rRNA was included in separate wells using VIC (TaqMan Assay) or primers as real-time reporter. The $\Delta\Delta C_T$ method was used to calculate the relative differences between experimental conditions and control groups as fold of change in gene expression. Details about the genes used in this study are provided in Supplemental Table 1 and Supplemental Table 2.

Liver total RNA and protein extract preparation

Total RNA from liver was isolated from 100 mg of frozen tissue using Trizol (#15596018, Life Technologies, Massachusetts, USA) and RNeasy Mini kit (Cat. No. 74104, Qiagen, Germany). The cDNA was synthesized from 1.5 μg of DNase-treated RNA. For total liver protein isolation, 100 mg of frozen tissue were homogenized in 50 mM HEPES pH=7.4, 1 mM EDTA, 2 mM EGTA with proteases and phosphatases inhibitors (1 mM PMSF, 2 mM NaF, 10 μg/mL leupeptin, 10 μg/mL aprotinin, 1 μg/mL pepstatin and 2 mM Na_3VO_4). The homogenate was centrifugated 10000 rpm for 10 min at 4°C to produce a total protein lysate. All extracts were stored at -80°C until use. Bradford protein assay was used for total protein quantification (#500-006, Bio-Rad, California, USA).

Western blot analysis and immunoblotting

Primary polyclonal antibodies were: anti-β-klotho (1:1000, PA5-21078) from Thermo Fisher; anti-pSer565-HSL (1:200, #4137), anti-phospho-p44/42-ERK1/2 (1:1000, #9106) and anti-p42/44-ERK1/2 (1:1000, #9102) from Cell Signaling Technology; anti-UCP1 (1:1000; sc-518024), and anti-β3-Adr (1:1000, sc-515763) from Santa Cruz Biotechnology; anti-FGF21-R1 (1:1000, ab156013), anti-FGF21 (1:1000, ab171941), anti-PGC-1a (1:1000, ab72230), anti-ATGL (1:200, ab109251), anti-HSL (1:1000, ab45422), anti-PPARβ/δ (1:1000, ab23676) and anti-β-actin (1:1000, ab8226) from Abcam, Cambridge, UK. Total OXPHOS rodent WB antibody cocktail (6µg/mL, ab110413) containing 5 mouse monoclonal antibodies: anti-CI subunit NDUFB8, anti-CII, anti-CIII-Core protein, anti-CIV subunit I and anti-CV alpha subunit was used from Abcam, Cambridge, UK.

The secondary antibody used were: goat anti-rabbit conjugated with horseradish peroxidase (1:4000, 172-1019) and goat anti-mouse conjugated with horseradish peroxidase (1:4000, 170-6516) from Bio-Rad, Spain. The immune-protein complexes formed were visualized using the ECL Western-blotting detection kit (Amersham Biosciences, Inc., Piscataway, NJ) and the images were subjected to a densitometric analysis.

Samples from experimental groups were compared in the same blots and routinely checked with loading controls (β-actin). The densitometric values of p565-HSL and phospho-p44/42-ERK1/2 were normalized with the densitometric values of the corresponding amount of total protein mass of HSL and 44/42-ERK1/2, respectively, in the same sample. The densitometric values of phospho-44/42-ERK1/2 were normalized with the densitometric values of the red ponceau assay due to similar size of the target protein with loading control. Data were expressed as a ratio of phosphor-44/42-ERK1/2/p44/42-ERK1/2 and p565-HSL/HSL.

Supplemental Table 1. Probes used for real time PCR.

Gene	ABI Assay ID
Arg-1	Rn00691090_m1
β-Klotho	Rn01413116_m1
Ccl5	Rn00579590_m1
Crh	Rn01462137_m1
Cpt-1α	Rn00580702_m1
Fgf21	RN00590706_m1
Fgfr1	Rn00577234_m1
Fgfr2	Rn01269940_m1
Fgfr3	Rn00584799_m1
Fgfr4	Rn01441815_m1
Glut4	Rn00562597_m1
Nrf2	Rn01767215_m1
Npy	Rn00561681_m1
Pgc-1α	Rn00580241_m1
P2rx5	Rn00589966_m1
Prdm16	Rn01516224_m1
Pparα	Rn00566193_m1
Pparβ/δ	Rn00565707_m1
Pparγ	Rn00440945_m1
Pomc	Rn00595020_m1
Tbx15	Rn01533809_m1
Ucp-1	Rn00562126_m1
18Sr RNA	4319413E

Supplemental Table 2. Primer sequences used for real time PCR.

Gene	Forward Sequence (5'—3')	Reverse Sequence (5'—3')
ObRb	TGGATAAACCCTTGCTCTTCA	GCATGCAGAATCACTGATATTTGG
Leptin (ob)	TCACCCCATTCTGAGTTTTGTC	CGCCATCCAGGCTCTCT
Trh	AGCTCAGCATCTTGGAAAGC	CCAGCAGCAACCAAGGTC
18Sr RNA	CGGCTACCACATCCAAGGAA	GCTGGAATTACCGCGGCT

Supplemental Table 3. Effects of central leptin treatment on the expression of Ob-Rb, Pomc, Crh and Trh in the hypothalamus.

	SS	PF	Lep
**Ob-Rb**	0.99±0.29[a]	2.73±0.17[b]	3.71±0.38[c]
**Pomc**	1.07±0.09[a]	0.39±0.02[b]	0.65±0.02[c]
**Crh**	0.88±0.09[a]	0.59±0.07[b]	1.96±0.10[c]
**Trh**	1.08±0.08[a]	0.89±0.07[a]	2.59±0.33[b]

mRNA relative levels of Ob-Rb, Pomc, Crh and Trh in hypothalamus. Data were obtained from 6 animals per group. The mean ± SEM is represented. The different letters show significant differences (p≤0.05) between groups, using a one-way ANOVA following by Tukey test. Groups: Saline: rats infused with the vehicle (PBS) and fed *ad libitum*; PF: rats infused with the vehicle and fed with the leptin group; Leptin: rats infused with leptin and fed *ad libitum*.

Supplemental Figure S1

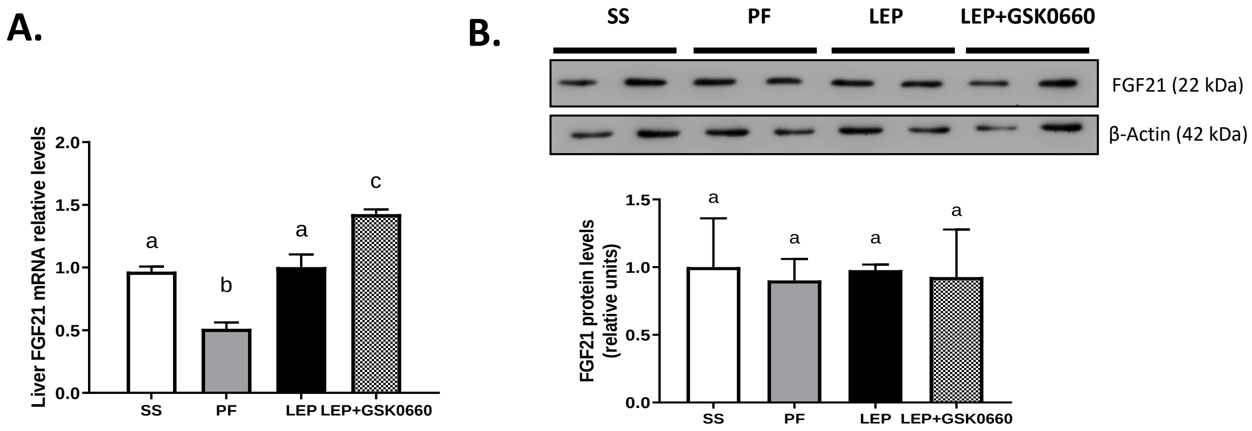

Supplemental Figure S1. A) mRNA relative levels of *Fgf21* in liver of rats after 7 days of chronic central treatment of vehicle (SS and PF), leptin and, leptin and ip GSK0660 co-administration (n=4-6). B) FGF21 total protein content in liver of rats after 7 days of chronic central treatment of vehicle (SS and PF, leptin and, leptin and ip GSK0660 co-administration (n=4-6). Data from real-time RT-PCR are expressed relative to 18S rRNA and the mRNA levels are expressed relative to SS group, the level of which was set at 1.0 arbitrarily. In Western-Blot analysis, densitometric levels of proteins are expressed relative to the SS group, the level of which was set at 1.0 arbitrarily. Results are the mean ± SEM per group of animals. Significant differences between ICV-treated rats were analyzed by One-Way ANOVA followed by Tukey test (differences letters mean significant differences, p≤0.05). SS: vehicle-infused rats fed *ad libitum*, PF: vehicle-infused pair-fed rats; Lep: leptin-infused rats; Lep+GSK0660: leptin-infused rats plus PPARβ/δ antagonist GSK0660.
